# The Efficacy of Divabirth Vaginal Dilator to Prevent Pelvic Floor Trauma During Labor: A Protocol Study

**DOI:** 10.29337/ijsp.179

**Published:** 2022-10-28

**Authors:** Muhammad Nurhadi Rahman, Siswanto Agus Wilopo, Ova Emilia

**Affiliations:** 1 Department of Obstetrics and Gynecology, Faculty of Medicine, Public Health, and Nursing, Universitas Gadjah Mada/Dr. Sardjito Hospital, Yogyakarta, Indonesia, 55281, ID; 2 Department of Biostatistics, Epidemiology and Population Health, Faculty of Medicine, Public Health, and Nursing, Universitas Gadjah Mada, Yogyakarta, Indonesia 55281, ID

**Keywords:** vaginal dilator, labor, protocol, perineal laceration, outcomes

## Abstract

**Background:**

Vaginal birth may have a negative impact on nerve structure and function, pelvic floor muscle structure, and function. Reducing the risk of pelvic floor injuries during vaginal birth is one of the most effective ways to prevent labor morbidity in women. There is a lack of tools developed based on this approach, especially in Indonesia. Therefore, we aimed to know the efficacy of a vaginal dilator called Divabirth based on labor outcomes.

**Methods:**

This clinical study involved subjects who are randomly grouped in to the control and treatment groups. Subjects in the treatment group are told to utilize vaginal dilator devices for 20 minutes each session, a maximum of two sessions per day, lasting 5 minutes every cycle, from 35 weeks of gestation until delivery.

**Discussions:**

The current research contributes valuable information to developing a vaginal dilator intervention program for pregnant women to reduce perineal laceration and improve labor outcomes. It may also help to lower their medical and treatment expenditures. We expect its success to be a step forward in improving reproductive health status.

**Highlights:**

- Vaginal birth may have negative impact on nerve and pelvic floor muscle structure and function
- Lack of tool has been developed to reduce the risk of pelvic floor injuries during vaginal birth
- A study protocol of vaginal dilator usage to know the efficacy based on labor outcomes

## 1. Background

Cesarean delivery has a greater morbidity rate than vaginal birth, including higher transfusion rates, critical care, uterine rupture, risk of hysterectomy, postpartum infection, and maternal mortality [1,2]. According to the most recent statistics from 150 countries, cesarean section (SC) births account for 18.6% of deliveries worldwide). Latin America and the Caribbean had the highest SC rate (40.5%), while Africa had the lowest (7.3%). Based on statistics from 1990 to 2014, there was a 12.4% rise in SC delivery (from 6.7% to 19.1%), with an average yearly increase of 4.4% [3]. This increase might be attributed to various benefits of CS birth, such as a decreased chance of urine incontinence and pelvic organ prolapse [4,5].

The literature indicating the association between vaginal birth and injuries to pelvic tissues has been well-researched. Vaginal birth may have a negative impact on nerve structure and function, pelvic floor muscle structure and function, pelvic organ support, and external anal sphincter structure and function. The longer the delivery, the higher the risk of pelvic anatomical structure and function impairment [6,7].

Reducing the risk of pelvic floor injuries during vaginal birth is one of the most effective ways to prevent labor morbidity in women. Few tools have been developed based on this approach, and they are still infrequently utilized. As a result, the current research aims to develop an intervention program based on the effects of vaginal dilators on labor outcomes.

### Divabirth

Divabirth is a vaginal dilator device used to reduce the risk of pelvic floor injury during vaginal delivery. It contains a balloon that will be inserted into the vagina and inflated with the air to give pressure and train the pelvic floor’s flexibility. This device is originally manufactured by the local industries and uses materials that originate from Indonesia.

## 2. Aim

This clinical research has main and secondary outcomes, which are:

- Main Outcome
  1. Knowing the efficacy of using Divabirth vaginal dilator compared to non-using Divabirth to reduce perineal injuries during vaginal delivery.
- Secondary Outcome
  1. Comparing the efficacy of using and non-using Divabirth to prevent the perineal lacerations during the vaginal delivery.
  2. Comparing the efficacy of using and non-using Divabirth to prevent the severe perineal lacerations during the vaginal delivery.
  3. Comparing the efficacy of using and non-using Divabirth to prevent the episiotomy procedure during vaginal delivery.
  4. Comparing the efficacy of using and non-using Divabirth to prevent the avulsion of levator ani muscle during vaginal delivery.
  5. Comparing the efficacy of using and non-using Divabirth to prevent the ballooning during vaginal delivery.

## 3. Methods

This study is a prospective, randomized, and multicenter clinical trial to determine the efficacy of Divabirth as a vaginal dilator. Before the clinical study started, we created a prototype of a vaginal dilator product called Divabirth which PT Swayasa Prakarsa then produces based on good manufacturing practices. In this clinical study, we collected 142 subjects randomly divided into control and treatment groups using block randomization. Subjects in the treatment group were requested to utilize vaginal dilator devices for 20 minutes each session, a maximum of two sessions per day, lasting 5 minutes every cycle, from 35 weeks of gestation until delivery. The balloon is introduced to two-thirds of its length in the vagina, then inflated to the amount of stretch feeling that is pleasant for the individual and maintained for each cycle. The balloon is subsequently removed with the mother’s help, such as crowning and delivering the baby’s head. Subjects were asked to increase the size of the balloon each day progressively. The diameter attained each session, frequency of usage, and any difficulties discovered are also reported. Following recruiting, individuals will be required to make monthly visits until delivery. Contamination was reduced in the control group by frequent follow-up and accurate recordings in the record book. The logbook is used to track each topic visit.

Both groups received routine obstetric care during the prenatal, intrapartum, and postpartum periods. Obstetricians and midwives were unaware of the topic group distinction. Episiotomy, perineal laceration, severe perineal laceration, fetal weight, biparietal diameter, head circumference, and length of the second stage of labor were among the delivery data acquired from observations while delivering the baby and postpartum examination. Further investigation was performed six weeks postpartum to assess the existence of levator ani muscle avulsion and ballooning. The study has been approved by the Medical and Health Research Ethics Committee of the Faculty of Medicine, Public Health and Nursing, Universitas Gadjah Mada. The registration number is KE/FK/1143/EC/2020 and renewed with KE/FK/1263/EC/2021. This study has also been registered in the Research Repository Faculty of Medicine, Public Health and Nursing, Universitas Gadjah Mada with the register number 202207122.

### Study environment and population

The intervention was conducted at the four private midwifery clinics in Sleman district, Special Region of Yogyakarta. The chosen sites have a high rate of vaginal delivery with minimum intervention or based on the normal delivery care protocol [13].

### Sample size calculation

The study’s intended participants were pregnant women aged 33–35 weeks who came to the associated midwifery clinic. The sample size (N = 142) was calculated using the statistical indices: d = 0.71, z (1–a/2) = 1.64, and z (1–b) = 0.84(those statistical indices were obtained from Pangastuti et al (2016)). Seventy-one participants from each group were evaluated, assuming a 10% drop.

### Sampling method

**Figure 1:**
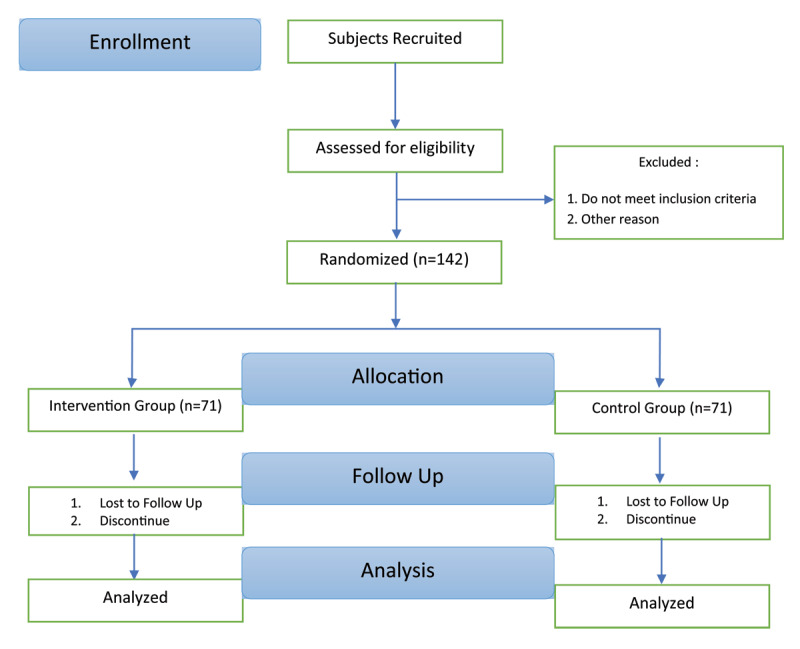
Study flowchart.

The technique of block randomization sampling was used to determine the participants. Participants will be randomly assigned to the intervention and control groups by an independent administrator using a computer-generated randomizer. Study flowchart can be seen in Figure 1.

### Inclusion criteria

The inclusion criteria were: (1) female patient with uncomplicated singleton pregnancy, (2) gestational age between 33–35 weeks, (3) aged ≥18 years, (4) never been pregnant for more than 20 weeks (primiparous), and (5) wish to undergo vaginal delivery.

### Exclusion criteria

The exclusion criteria were: multiparity, multiple pregnancies, unknown gestational age, estimated fetal weight >4000 g, pelvic anomalies, multiple sclerosis, collagen disease, placenta previa, previous vaginal or perineal surgery, acute or chronic vaginal infections, premature rupture of membranes, diabetic neuropathy, abuse drugs or alcohol, and paraplegia.

### Drop Out criteria

Subjects will be dropped out from the research if they experience a severe adverse event, lost to follow-up, and do not fulfill a complete follow-up session (four sessions).

### Data collection method

Participants were scheduled to have obstetrics and gynecological examinations at the midwifery clinic after signing the informed consent. They will be randomly assigned to the control and treatment groups based on the random blocks.

### Data analysis

The univariate analysis will determine the characteristics of the research subjects and compare the treatment and control groups by calculating the frequency and proportion in each group.

The bivariate analysis will determine the strength of the relationship between two variables, including independent variables with dependent variables and external variables with dependent variables without control. Comparative analysis of numerical variables between the two groups used the independent t-test statistical test if the data obtained a normal distribution or the Mann-Whitney U test if the distribution was not normal. Comparative analysis of the proportions of categorical variables between two groups used the Chi-Square or Fischer’s exact test. Multivariate logistic regression analysis was used to determine the relationship between independent and dependent variables. All analyses of our data used SPSS version 21 (IBM Corp., Armonk, NY) with 95% confidence interval (CI) and significance set as *p* < 0.05. Divabirth will be considered effective in reducing perineal injury compared to the control group if p < 0.05.

### Outcome measures

Perineal laceration of any degree, episiotomy, levator ani muscle avulsion, and ballooning are the maternal outcomes evaluated following the birth.

## 4. Discussion

Labor training tools in the form of vaginal dilators are another frequently utilized method of minimizing perineal trauma, and the most often used of which is EpiNo®. Kavvadias and Hoesli [8] observed that EpiNo® reduced the rates of episiotomy, perineal rupture, the risk of microtrauma, and levator ani avulsion but this effect was not always statistically significant.

In Germany, the EpiNo® vaginal dilator has been on the market since October of 1999. It comprises an inflating balloon attached to a manometer through a tube. The silicone balloon is formed like a figure eight and has two different ends that may be inflated between the narrower (waist) reinforced region. The balloon is inflated to 60 mm Hg and coated with lubricant before vaginal insertion. The balloon is placed into the vagina until 3 cm of the balloon is visible outside the vagina. The waist is located in the hymenal region. When the manometer is activated, the balloons are individually inflated, causing the woman to feel the enlargement. The patient then keeps the balloon in the vagina for at least 10 minutes before forcing it out using her abdominal and pelvic muscles. The patient will be able to effectively practice removing the balloon with a size that gradually matches the baby’s head by increasing the diameter of the balloon every day [9]. There are still disparities in study findings of vaginal ‘dilators’ ability to prevent pelvic floor damage.

A comprehensive evaluation of five clinical studies by Kavvadias and Hoesli [8] found that EpiNo® decreased episiotomy rates, perineal rupture, and the risk of microtrauma and levator ani avulsion; however, this was not always statistically significant. The gadget did not influence the length of the second stage of labor or the fate of the fetus. Because the device is well accepted and seldom produces adverse effects, it may be inferred that it can be effective for helping normal delivery, although further study is needed.

Nakamura et al. [10] discovered that EpiNo® induced relatively little pain (mean Visual Analogue Score 3.8) in 227 term women, with primiparas expressing more significant levels of discomfort than multiparas (4.5 vs. 3.1, respectively), *p* < 0.001). The results indicated a negative association (r = –0.424, *p* < 0.001), which means that the higher the distension caused by the device, the lower the reported discomfort.

Brito et al. [11] discovered that EpiNo® did not reduce the rate of episiotomy (RR = 0.92 [95% CI: 0.75–1.13], n = 710, *p* = 0.44; 2 trials; fixed model), second stage of labor (MD = –12.50 [95% CI: –29.62, –4.62], n = 162, *p* = 0.54; one test; fixed model), or the proportion of intact perineum (RR = EpiNo® had no impact in reducing all forms of perineal lacerations or grade lacerations (RR = 0.99 [95% CI: 0.84–1.17], n = 705, *p* = 0.93, two trials; fixed model), n = 705, *p* = 0.38, two tests; fixed model (RR = 1.31 [95% CI: 0.72–2.37], n = 705, *p* = 0.38, two tests; fixed model). In both trials, the mean birth weight of the EpiNo® group was greater than that of the control group, but the difference was not statistically significant.

A randomized clinical study [12] comparing EpiNo® and conventional care in 504 women in Australia revealed no significant difference in the frequency of levator avulsion, irreversible Hiatal over distance, anal sphincter damage, or perineal laceration between the intervention and control groups. The intervention group had a more significant external anal sphincter defect (RR = 1.44, 95% CI: 0.97–2.20).

The current study provides important information for developing a vaginal dilator for pregnant women to improve labor outcomes and reduce perineal laceration, especially in Indonesia.

It may also help to lower their medical and treatment expenditures. We believe that this device has the potential to be integrated into professional health care recommendations, assisting physicians and health care professionals in paying attention to the critical role of women›s health, particularly during labor and postpartum. The device may be vital and cost-effective, and we expect that its success will be a step forward in improving reproductive health status.

## Sponsors and Collaborators

This study is partially sponsored by the Faculty of Medicine, Public Health and Nursing, Gadjah Mada University in collaboration with PT Swayasa Prakarsa as the manufacturer.
